# Surface modification-mediated biodistribution of ^13^C-fullerene C_60_ in vivo

**DOI:** 10.1186/s12989-016-0126-8

**Published:** 2016-03-08

**Authors:** Chenglong Wang, Yitong Bai, Hongliang Li, Rong Liao, Jiaxin Li, Han Zhang, Xian Zhang, Sujuan Zhang, Sheng-Tao Yang, Xue-Ling Chang

**Affiliations:** 1 Northwest University, Xi’an, 710069 P. R. China; 2 CAS Key Laboratory for Biomedical Effects of Nanomaterials and Nanosafety, Institute of High Energy Physics, Chinese Academy of Sciences, Beijing, 100049 P. R. China; 3 College of Chemistry and Environment Protection Engineering, Southwest University for Nationalities, Chengdu, 610041 P. R. China; 4 Key Lab of Urban Environment and Health, Institute of Urban Environment, Chinese Academy of Sciences, Xiamen, 361021 P.R. China

**Keywords:** Fullerene, Hydroxylation, Carboxylation, Biodistribution, Isotopic labeling

## Abstract

**Background:**

Functionalization is believed to have a considerable impact on the biodistribution of fullerene in vivo. However, a direct comparison of differently functionalized fullerenes is required to prove the hypothesis. The purpose of this study was to investigate the influences of surface modification on the biodistribution of fullerene following its exposure via several routs of administration.

**Methods:**

^13^C skeleton-labeled fullerene C_60_ (^13^C-C_60_) was functionalized with carboxyl groups (^13^C-C_60_-COOH) or hydroxyl groups (^13^C-C_60_-OH). Male ICR mice (~25 g) were exposed to a single dose of 400 μg of ^13^C-C_60_-COOH or ^13^C-C_60_-OH in 200 μL of aqueous 0.9% NaCl solution by three different exposure pathways, including tail vein injection, gavage and intraperitoneal exposure. Tissue samples, including blood, heart, liver, spleen, stomach, kidneys, lungs, brain, large intestine, small intestine, muscle, bone and skin were subsequently collected, dissected, homogenized, lyophilized, and analyzed by isotope ratio mass spectrometry.

**Results:**

The liver, bone, muscle and skin were found to be the major target organs for C_60_-COOH and C_60_-OH after their intravenous injection, whereas unmodified C_60_ was mainly found in the liver, spleen and lung. The total uptakes in liver and spleen followed the order: C_60_ > > C_60_-COOH > C_60_-OH. The distribution rate over 24 h followed the order: C_60_ > C_60_-OH > C_60_-COOH. C_60_-COOH and C_60_-OH were both cleared from the body at 7 d post exposure. C_60_-COOH was absorbed in the gastrointestinal tract following gavage exposure and distributed into the heart, liver, spleen, stomach, lungs, intestine and bone tissues. The translocation of C_60_-OH was more widespread than that of C_60_-COOH after intraperitoneal injection.

**Conclusions:**

The **s**urface modification of fullerene C_60_ led to a decreased in its accumulation level and distribution rate, as well as altering its target organs. These results therefore demonstrate that the chemical functionalization of fullerene had a significant impact on its translocation and biodistribution properties. Further surface modifications could therefore be used to reduce the toxicity of C_60_ and improve its biocompatibility, which would be beneficial for biomedical applications.

## Background

The physicochemical properties of nanomaterials, including their size, shape, aspect ratio, charge, composition, surface chemistry and exposure route, may have a significant impact on their molecular toxicity, cellular uptake, transport/fate, absorption, distribution, metabolism, excretion and toxicity in living organisms, thereby affecting their applications in biomedicine [1–6]. The surface modification of nanomaterials plays an essential role in improving their biocompatibility, regulating their in vivo distribution and metabolism, and reducing their toxicity in the environment, which is especially important for poorly soluble or insoluble nanomaterials (e.g., carbon nanomaterials) [6–8]. Research directed toward exploring the surface chemistries of carbon nanomaterials can be used to develop a better understanding of their novel properties and functions for biomedical and numerous other applications [9–11]. For example, the water-soluble derivatives of C_60_ have been developed for cancer treatment, antioxidation against oxidative stress and free radicals, and bioimaging, etc. in biomedical applications [9–11]. However, the unique properties of nanomaterials may also pose unexpected hazards to human health and the environment [12–15]. The development of simple, reliable and stable analytical methods for the quantitative in vivo detection of nanomaterials is therefore critical to evaluate their biosafety and provide information concerning nanofactor-relevant biological effects and toxicity [16, 17]. Quantitative data pertaining to the characteristics of nanomaterials may be beneficial for designing safe and effective nanomaterials/nanodrugs, establishing the predictive models of nanomaterials exposure, refining the toxicological theories, developing a thorough understanding of nanotoxicology mechanisms and regulating risk management strategies for nanomaterials [18, 19].

The development of efficient methods for the quantification of carbon nanomaterials in vivo is quite rightly regarded as the first and most important step in evaluating their biosafety [16]. The biodistribution of carbon nanomaterials has been investigated extensively using a diverse variety of quantification techniques [20]. Based on a comparison of the results generated to date by these different groups, it has been possible to devise several guidelines concerning the pharmacokinetic and biodistribution properties of carbon nanomaterials, which have been communicated to the wider scientific community [16, 20–24]. Among them, the surface functionalization of carbon nanomaterials is believed to have a significant influence on their biodistribution properties. For example, pristine carbon nanotubes (CNTs) and PEGylated CNTs showed completely different blood circulation and reticuloendothelial system (RES) accumulation levels after intravenous administration [25–29].

In light of the difficulties associated with the standardization of carbon nanomaterials, one could be forgiven for viewing proposed regularities in the biodistribution patterns of carbon nanomaterials obtained by comparing the results from different groups with some suspicion [16]. Even identical materials prepared by different groups using the same protocol can generate very different biodistribution data and considerable differences may also be observed during the evaluation of otherwise identical carbon nanomaterials by different labs. For instance, Singh et al. and McDevitt et al. [30–33] independently reported the preparation of amino-CNTs using the same protocol. While Singh et al. reported that the amino-CNTs were rapidly cleared from the body, McDevitt et al. reported much longer retention in RES organs. There is therefore an urgent need for the development of an analytical method for the direct quantitative comparison of the biodistribution profiles of differently functionalized carbon nanomaterials to address these issues.

Fullerene C_60_ has a well-defined structure that can be reliably quantified by stable isotope labeling [34, 35]. Stable isotope labelling avoids some of the drawbacks associated with radioactive labeling, such as the requirement for radioactive operating conditions and the generation of radioactive wastes. Furthermore, the introduction of radioactive or fluorophore tags can lead to pronounced changes in the surface chemistry of nanomaterials, thereby altering their transport and pharmacokinetic properties in vivo. In contrast, stable isotope ^13^C skeleton-labeled fullerene retains the intrinsic properties of normal fullerene and its preparation does not require a radioactive protocol. Moreover, stable isotopic tracers can be used to readily distinguish between the endogenous and exogenous sources of elemental building blocks. Stable isotopic tracers are also beneficial for differentiating between ultrafine carbon particles, carbon nanomaterials and carbon-rich biological samples with similar compositions and dimensions [36–41]. The natural abundance of ^13^C is 1.1% (atom ratio), which results in high background ^13^C levels. However, the ratio of ^13^C to ^12^C is constant in all living organisms, which means that it does not interfere with the precision and accuracy of the test. Stable ^13^C has been incorporated into the carbon skeletons of numerous carbon nanomaterials, and the transportation and transformations profiles of the resulting isotope-labeled materials were readily detected by isotope ratio mass spectrometry (IRMS) [25, 29, 35, 42]. The enrichment or depletion of ^13^C in a sample can be expressed using the delta notation (*δ* ^13^C) to a high level of precision (within ±0.1‰) [43, 44]. The quantitative comparison of differently functionalized ^13^C-C_60_ materials could therefore provide a definitive understanding of the role played by functionalization in the biodistribution of carbon nanomaterials.

**Fig. 1:**
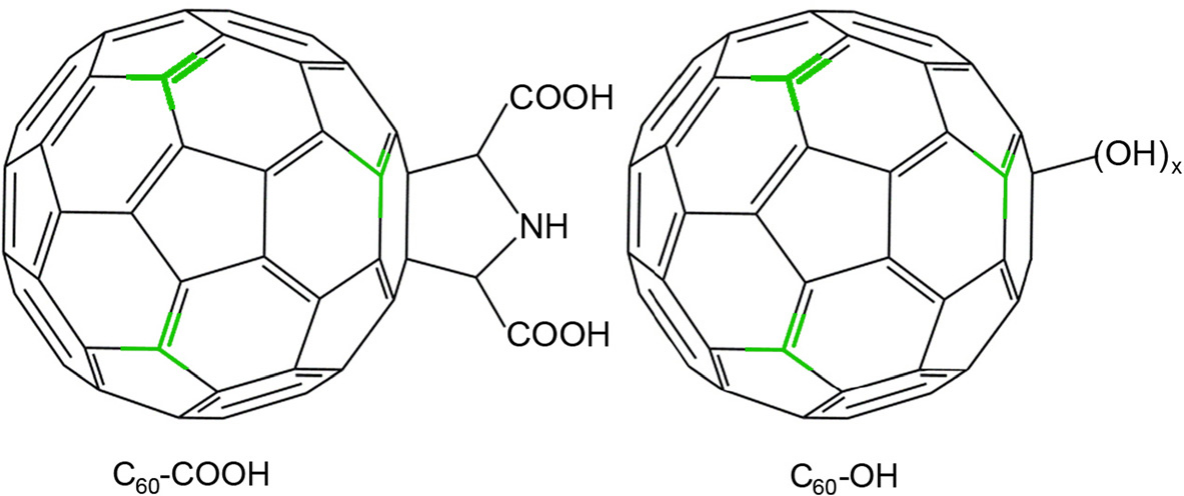
Structure of ^13^C-C_60_-COOH (left) and ^13^C-C_60_-OH (right). The ^13^C atoms (5 ~ 6 atoms per fullerene cage) were highlighted in green

The primary aim of our study was to reveal the influence of surface functionalization on the biodistribution of fullerene. In this study, we have prepared ^13^C skeleton-labeled fullerene (i.e., ^13^C-C_60_) materials functionalized with carboxyl groups (^13^C-C_60_-COOH) or hydroxyl groups (^13^C-C_60_-OH) (Fig. 1) and compared their in vivo biodistribution profiles following their administrations via three different exposure pathways. C_60_-COOH and C_60_-OH showed differences in their biodistribution and body clearance profiles after intravenous (i.v.) injection. C_60_-COOH was mostly absorbed in the gastrointestinal tract, whereas C_60_-OH was translocated throughout a variety of different tissue types after intraperitoneal (i.p.) injection. The results of this study have provided conclusive evidence that the chemical functionalization of carbon nanomaterials has a significant impact on their translocation and biodistribution behaviors.

**Fig. 2:**
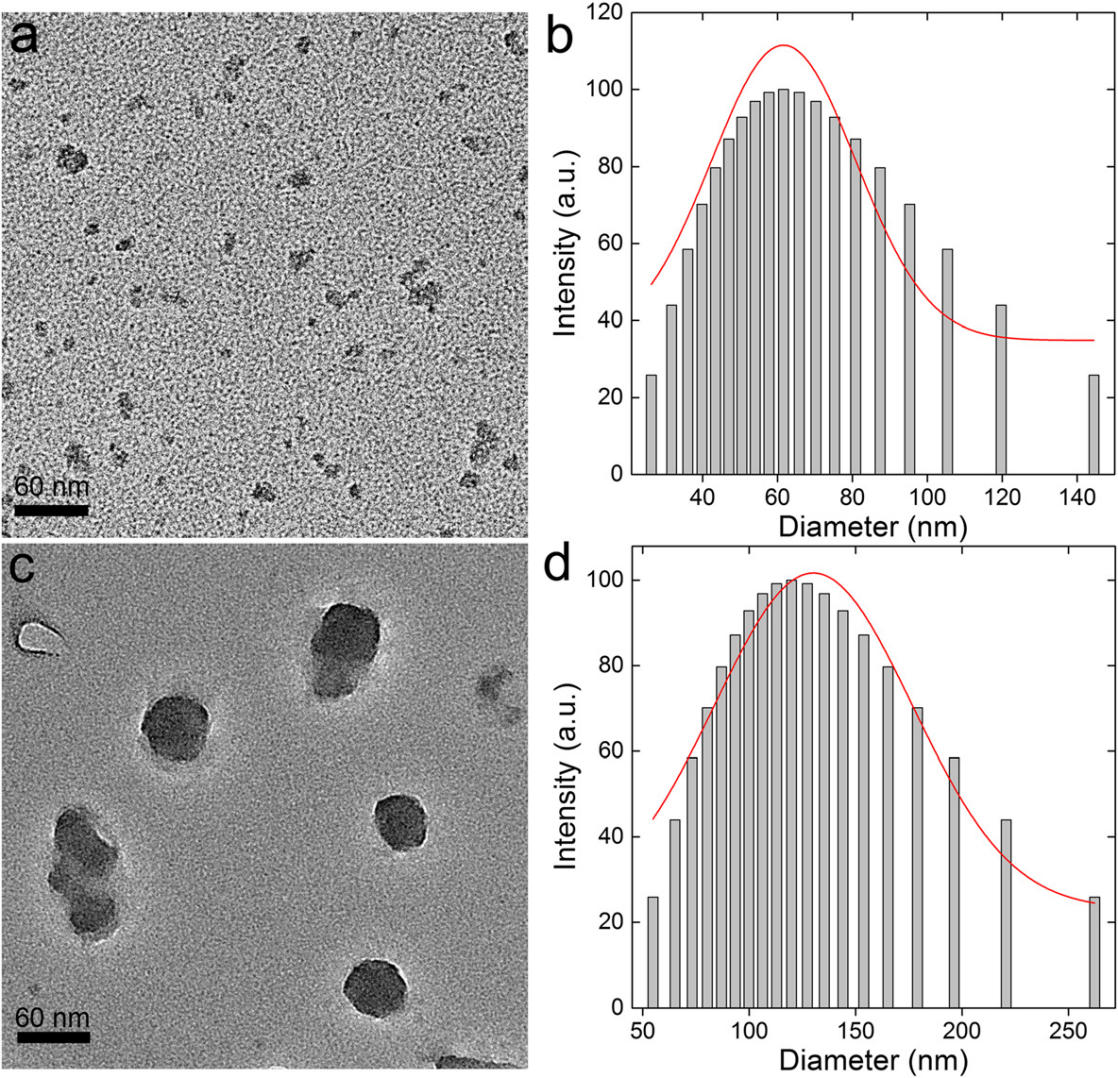
Representative TEM images (**a**, **c**) and size distribution patterns (**b**, **d**) of C_60_-COOH (**a**, **b**) and C_60_-OH (**c**, **d**)

**Fig. 3:**
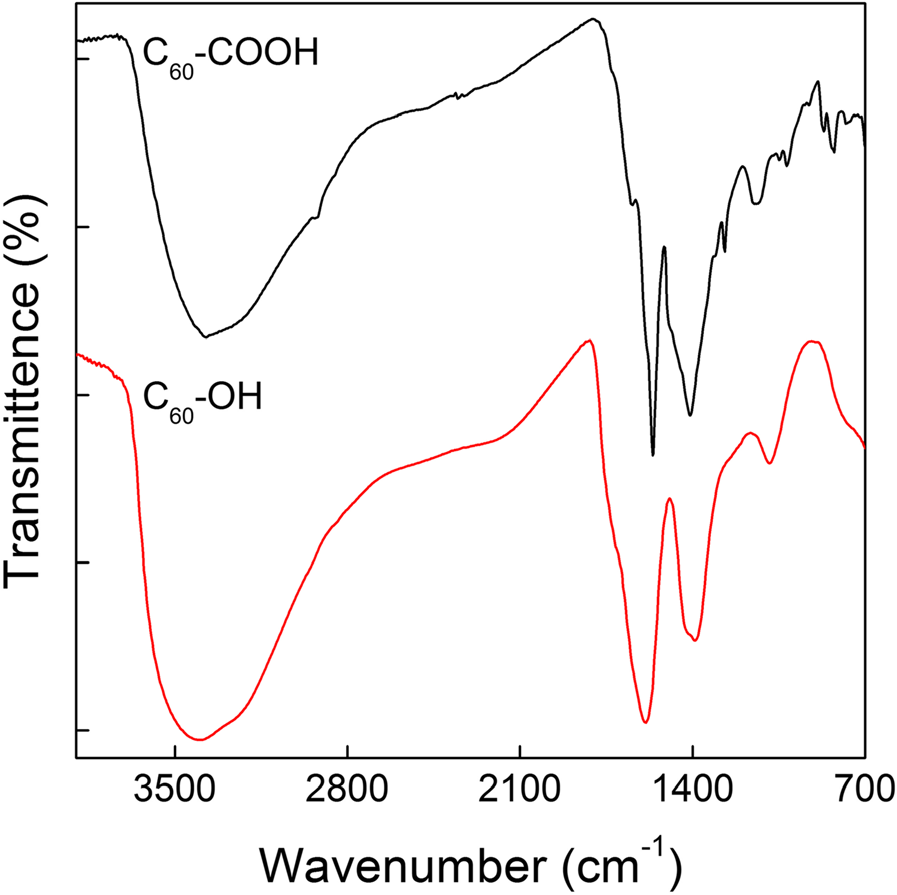
IR spectra of C_60_-COOH (black line) and C_60_-OH (red line)

**Fig. 4:**
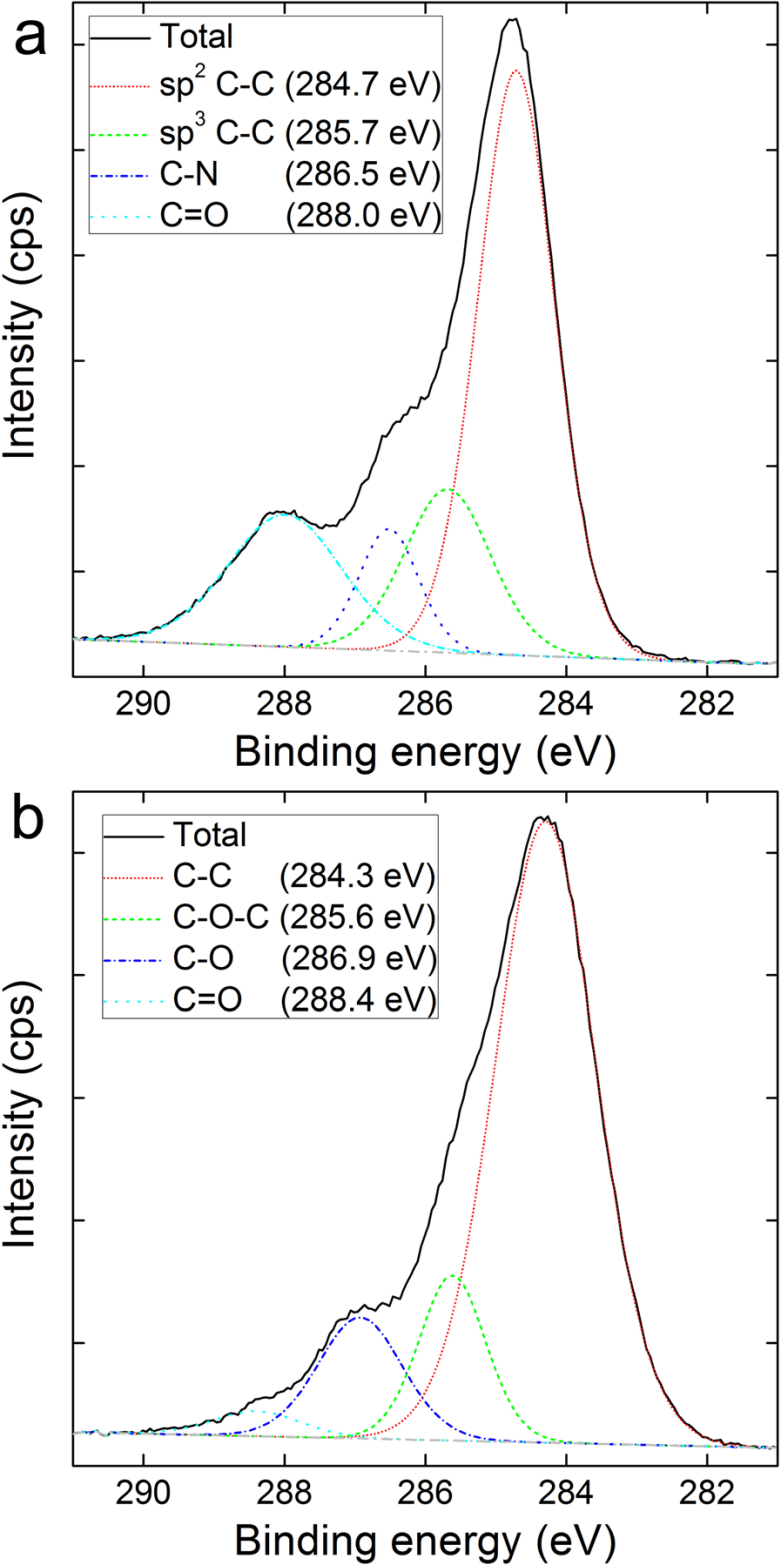
Biodistribution of C_60_-COOH (red columns) and C_60_-OH (blue columns) in mice after i.v. injection at different time points. ^*^ *p* < 0.05 compared between C_60_-COOH and C_60_-OH

**Fig. 5:**
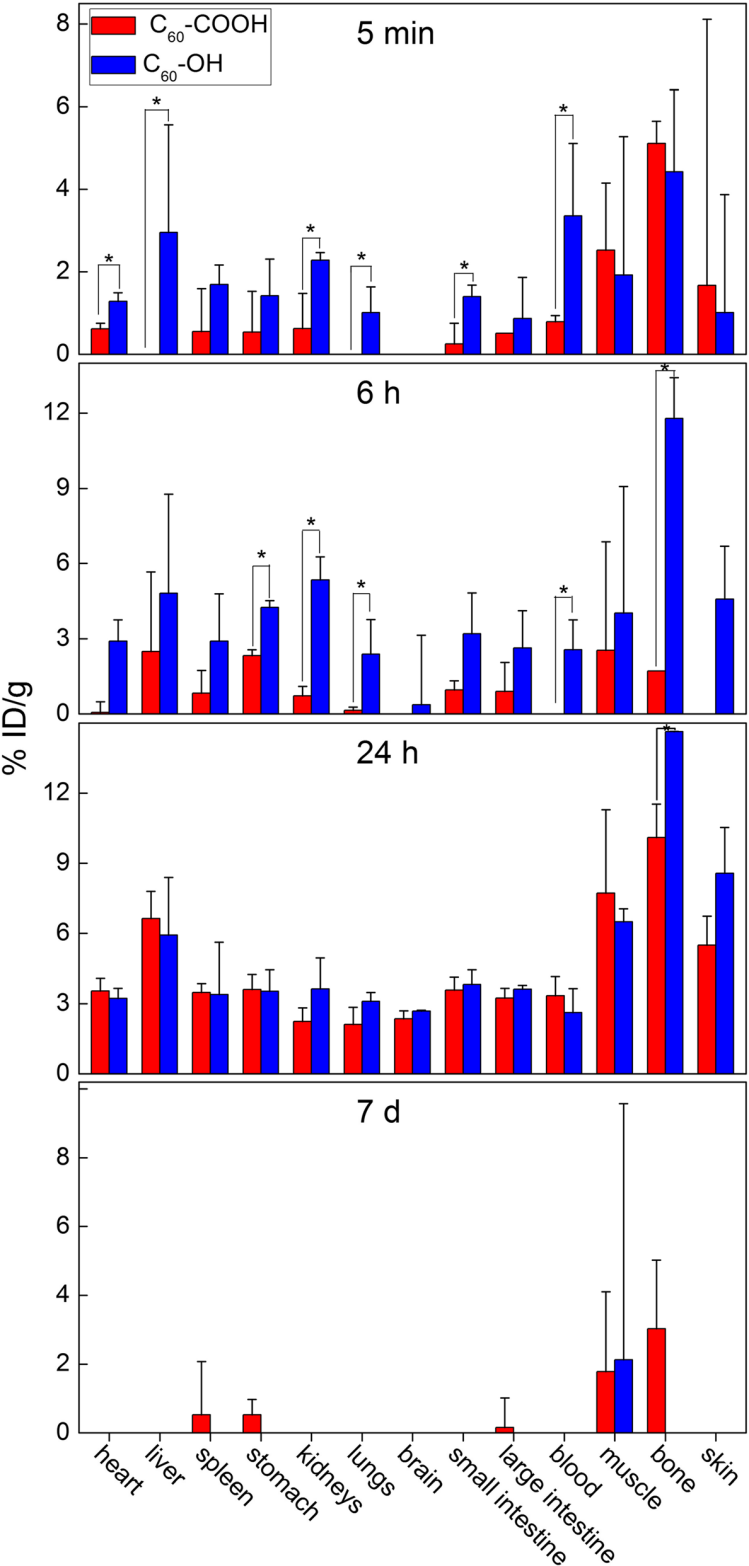
C1s XPS spectra of C -COOH (a) and C -OH (b)

**Fig. 6:**
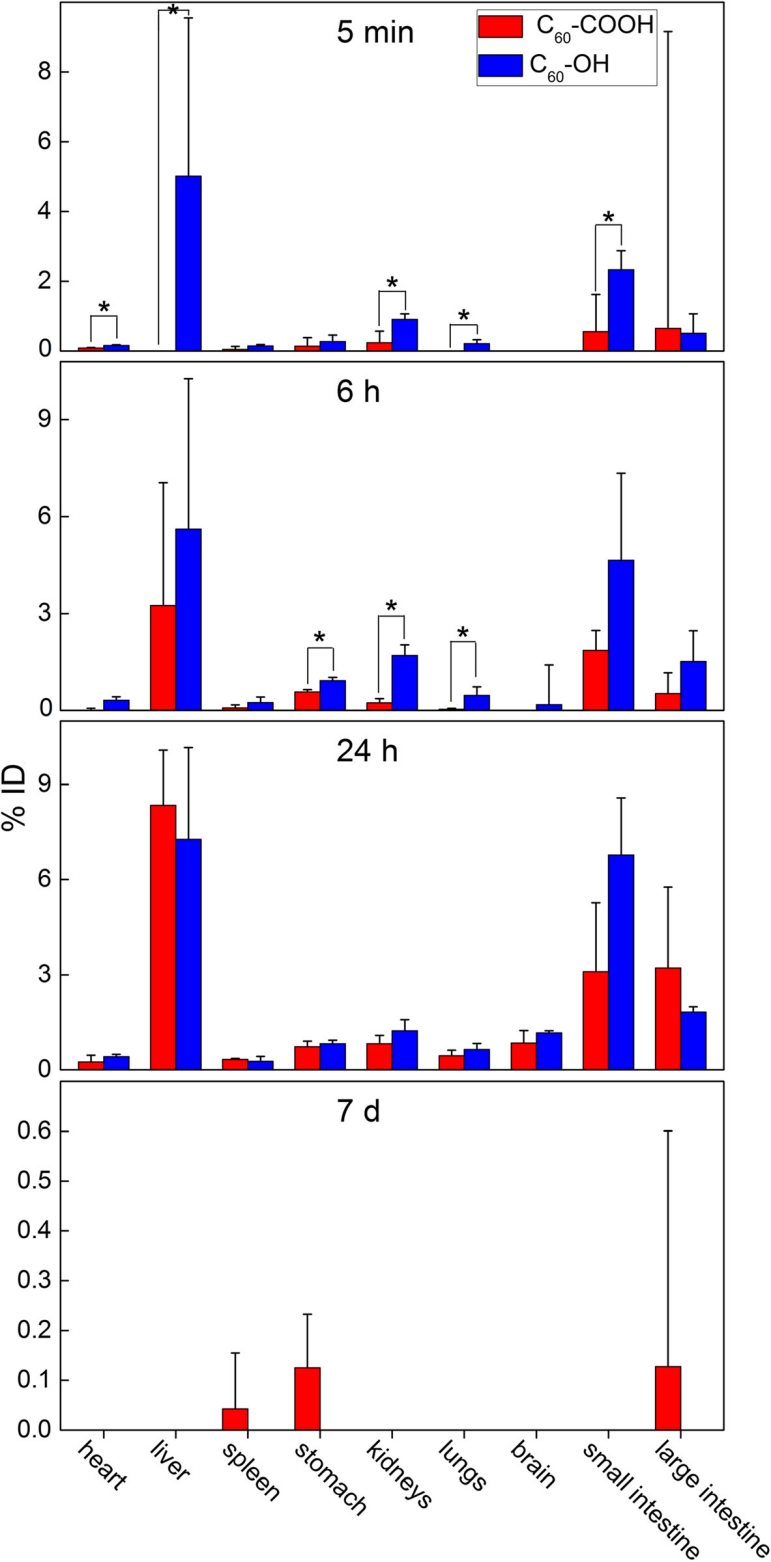
Uptakes of C_60_-COOH (red columns) and C_60_-OH (blue columns) in organs after i.v. injection at different time points. ^*^ *p* < 0.05 compared between C_60_-COOH and C_60_-OH

**Fig. 7:**
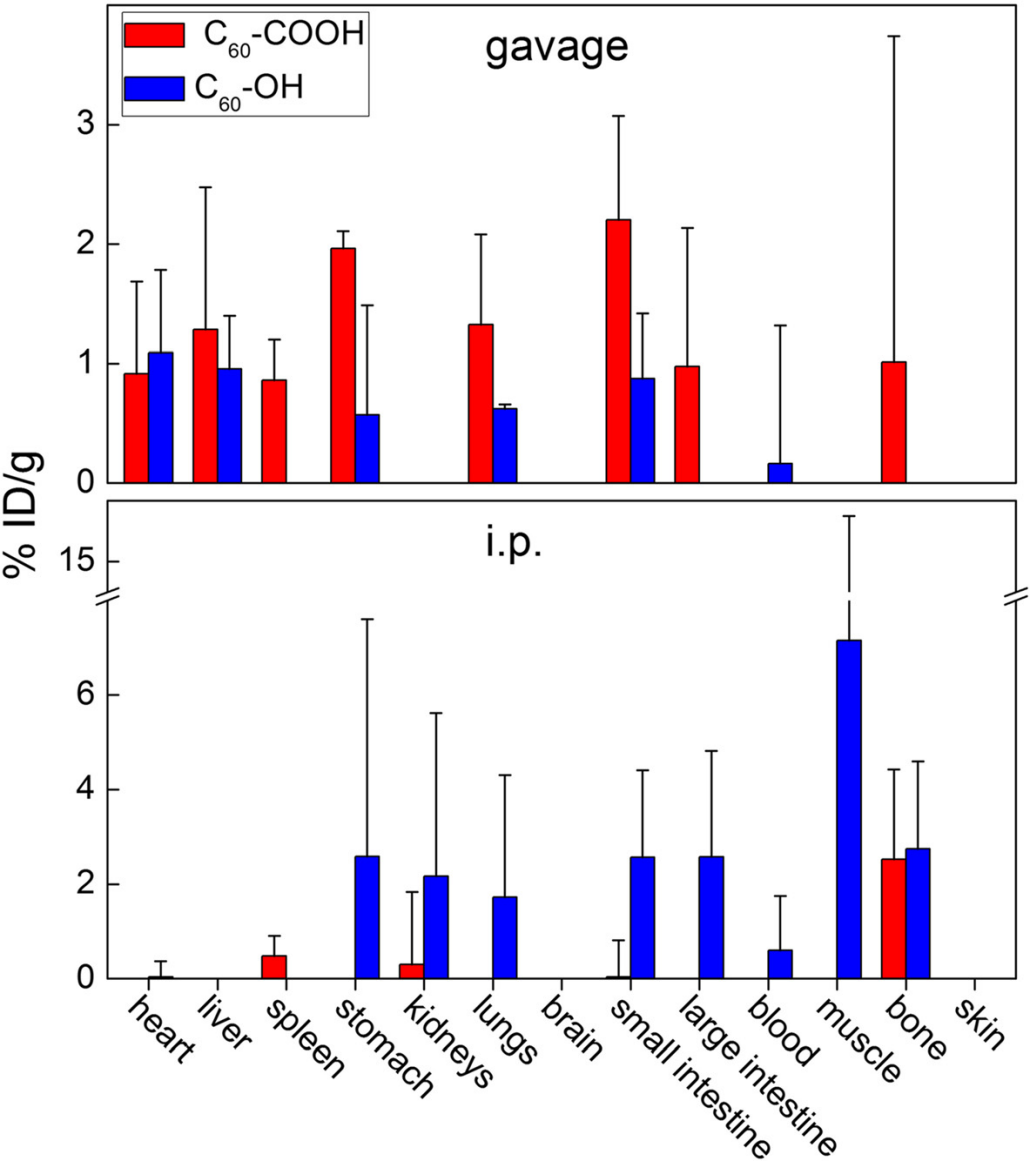
Translocation and biodistribution of C_60_-COOH (red columns) and C_60_-OH (blue columns) in mice at 24 h after gavage and i.p. exposures

## Results

### Characterization of C_60_-COOH and C_60_-OH

C_60_-COOH and C_60_-OH were both found to be highly dispersible in water and other aqueous systems. Furthermore, brown dispersions of C_60_-COOH and C_60_-OH were found to be stable for more than 6 months under ambient conditions without any visible precipitation. However, transmission electron microscopy (TEM) images revealed that very small aggregates were formed in aqueous solutions of C_60_-OH and C_60_-COOH, with diameters of 50 and 6–20 nm, respectively (Fig. 2). The size distribution patterns of both samples in solution are shown in Fig. 2b and d. The diameters of the C_60_-OH and C_60_-COOH particles in water were around 62 and 120 nm, respectively. The hydrodynamic radii of both samples were larger than the diameters observed in the TEM images.

The infrared (IR) spectra of C_60_-COOH and C_60_-OH were also collected to confirm that the functionalization reactions had proceeded successfully (Fig. 3). The IR spectrum of C_60_-COOH contained a broad peak at 3375 cm^−1^, which was attributed to the stretching vibrations of the -COOH/-NH- groups. A tiny peak was also observed at 2931 cm^−1^, which was attributed to the stretching vibrations of the -CH_2_- groups. The peaks at 1644 and 1560 cm^−1^ were assigned to the stretching vibrations of the C = O and C = C moieties, respectively. Last, the characteristic peak of the -COOH groups was observed at 1410 cm^−1^. The IR spectrum of C_60_-OH contained a broad peak at 3406 cm^−1^ for the -OH groups, an intense peak at 1588 cm^−1^ for the C = C groups and a characteristic peak corresponding to the bending vibrations of the -OH groups at 1393 cm^−1^. The differences in functionalities between C_60_-COOH and C_60_-OH were also evaluated by X-ray photoelectron spectroscopy (XPS). As shown in Fig. 4, the C1s XPS spectrum of C_60_-COOH contained a strong C = O peak at 288.0 eV, whereas the spectrum of C_60_-OH contained a very weak C = O peak at 288.4 eV. The appearance of C-O-C (285.6 eV) is a common phenomenon for C_60_-OH. XPS demonstrated the chemical state of carbon atoms. The lack of C-O in C_60_-COOH indicated that there was no -OH in C_60_-COOH. Similarly, the very weak C = O in C_60_-OH indicated that there was very limited or no -COOH in C_60_-OH.

The ^13^C abundances of ^13^C-C_60_-COOH and ^13^C-C_60_-OH were measured by IRMS. Urea was used as a working standard to calibrate the IRMS facility, which gave a *δ* ^13^C value of about -40.5573 ± 0.01106. The value determined for urea by IRMS was found to be equal to that of the certified value for urea, indicating that our IRMS system could be used to make reliable measurements. The *δ* ^13^C values for ^13^C-C_60_-COOH and ^13^C-C_60_-OH were determined to be 7910.69 and 7914.92, respectively. It is noteworthy that the *δ* values for ^13^C-C_60_-COOH and ^13^C-C_60_-OH were much higher than that of the urea standard value. Based on the equation for determining the delta (*δ*) parameter [35, 43–45], the ^13^C abundances (the atom percentage of ^13^C atoms per all carbon atoms in a specific fullerene derivative) were calculated to be about 8.204% ± 0.013% for ^13^C-C_60_-COOH and 8.310% ± 0.017% for ^13^C-C_60_-OH, equaling to 5.3 ^13^C-atoms per one ^13^C-C_60_-COOH and 5.0 ^13^C-atoms per one ^13^C-C_60_-OH. The atom percentage of ^13^C reported for the unmodified C_60_ in Ref. 35 was 8.752%. Collectively, there were about 5 ~ 6 ^13^C atoms per C_60_ cage, where the ^13^C atoms were incorporated in the skeleton of fullerene cage (Fig. 1). The amount of ^13^C found in the two fullerene nanomaterials was around 9.0%, which is higher than the weight percentage of ^13^C in natural carbon (1.2% ± 0.0013%). All of these characterization data indicated that the C_60_ derivative samples were suitable for the following in vivo quantification experiments.

### Biodistributions of C_60_-COOH and C_60_-OH after i.v. injection

The biodistributions of C_60_-COOH and C_60_-OH were studied following their i.v. injection in mice. C_60_-COOH and C_60_-OH both showed low accumulation levels (expressed as the percentage of the injected dose per gram of tissue, %ID/g) in the bodies of the mice (Fig. 5). The distributions of C_60_-COOH and C_60_-OH were detected in the whole body with relatively high levels in the liver, bone, muscle and skin tissues. The values obtained for the accumulation of C_60_-COOH and C_60_-OH in the RES organs in the current study were much lower than those reported in our previous study for unmodified C_60_ (liver: 15.3%ID/g; spleen: 63.5%ID/g; lung: 18.2%ID/g) [35]. Furthermore, the value for C_60_-COOH was slightly higher than that of C_60_-OH. During the first 5 min, it was clear that the distribution rate and accumulation levels of C_60_-OH were higher than those of C_60_-COOH. Considerable differences were observed in the accumulation levels of C_60_-COOH and C_60_-OH in the heart, liver, kidney, lung, small intestine and blood tissue samples. Furthermore, higher levels of accumulation were observed in the muscle and bone tissues for both C_60_-COOH and C_60_-OH compared with the other organs. The accumulation levels of C_60_-COOH and C_60_-OH increased over time with the accumulation levels of C_60_-COOH eventually catching up to those of C_60_-OH. At 24 h, we observed almost identical levels of trapping for C_60_-COOH and C_60_-OH, with very similar distribution patterns. C_60_-COOH resulted in slightly higher levels of accumulation in the liver compared with C_60_-OH following i.v. administration, whereas C_60_-OH resulted in higher accumulation levels in the bone and skin tissues. However, the only significant differences observed between the accumulation levels of C_60_-COOH and C_60_-OH were found in the bone. C_60_-COOH and C_60_-OH were almost completely eliminated from the test tissue samples at 7 d post exposure. Only low accumulation levels were detected in some of the tissues at this time point with no significant differences between the difference materials and tissues (*p* > 0.05 compared with the control group).

By multiplying the %ID/g data by the weights of the organs or tissues, it was possible to obtain the corresponding %ID (percentage of injected dose) data (Fig. 6). The total uptakes in the liver and spleen were of the order C_60_ > > C_60_-COOH > C_60_-OH. The distribution rate at 24 h followed the order C_60_ > C_60_-OH > C_60_-COOH. The liver and intestine were found to be the major target organs for C_60_-COOH and C_60_-OH at 24 h after i.v. injection, whereas unmodified C_60_ was mainly found in the liver (23.75%ID), spleen (17.85%ID) and lung (4.56%ID) [35]. It is noteworthy that the uptakes of C_60_-COOH and C_60_-OH in bone and muscle were found to be very large at 24 h, considering that the total weights of the bone and muscle tissues were high. However, we did not obtain the %ID values for the bone and muscle tissues because it was not possible to accurately determine the weights of the bone and muscle tissues and it would be unreasonable to infer that the accumulation levels were the same in all of the bone and muscle tissues.

The uptake levels in the liver, spleen and lung tissues were further analyzed to reveal the accumulations of C_60_-COOH and C_60_-OH in the RES organs. A comparison of the RES uptakes of C_60_-COOH, C_60_-OH and unmodified C_60_ is shown in Table 1 [35]. These results clearly show that the RES uptakes of the C_60_ derivatives decreased considerably following their functionalization. The uptake levels in the liver and spleen followed the order C_60_ > > C_60_-COOH > C_60_-OH. C_60_-COOH (9.1%ID) and C_60_-OH (8.2%ID) showed much lower RES uptakes than C_60_ (46.0%ID) [35]. Quantitatively, the hepatic accumulation of C_60_-COOH (8.3%ID) was found to be slightly higher than that of C_60_-OH (7.3%ID), whereas the spleen uptakes of C_60_-COOH (0.33%ID) and C_60_-OH (0.27%ID) were nearly the same. The level of C_60_-COOH accumulation (0.44%ID) in lung was slightly lower than that of C_60_-OH (0.64%ID), with both values being very low. According to the Student *t-*test, the differences observed between the unmodified C_60_ material and the soluble derivatives at 24 h post exposure were significant, suggesting that these surface modifications had altered the RES uptake of fullerene.

**Table 1:** Uptakes of C_60_-COOH, C_60_-OH and unmodified C_60_ in RES organs after i.v. injection 24 h

	C_60_-COOH (%ID)	C_60_-OH (%ID)	Unmodified C_60_ (%ID) ^a^
Liver	8.35 ± 2.37	7.27 ± 1.26	23.75 ± 3.81^*,§^
Spleen	0.33 ± 0.01	0.27 ± 0.10	17.85 ± 4.35^*,§^
Lung	0.44 ± 0.23	0.64 ± 0.23	4.56 ± 1.96^*,§^

^a^ The data of unmodified C_60_ are from our previous study in reference [35]. ^*^ *p* < 0.05 between C_60_-COOH and unmodified C_60_; ^§^ *p* < 0.05 between C_60_-OH and unmodified C_60_

### Biodistributions of C_60_-COOH and C_60_-OH after gavage and i.p. injection

The absorption and translocation properties of C_60_-COOH and C_60_-OH varied considerably depending on their route of administration (Fig. 7). For example, the results revealed that C_60_-COOH showed high levels of tissue accumulation following gavage administration, with high concentrations of C_60_-COOH observed in the stomach and intestine. Several other organs, including the heart, liver, spleen, lung and bone, were also targeted by this material. In contrast, the accumulation levels for C_60_-OH were all rather low at less than 1.0%ID/g. However, these differences in the accumulation levels of C_60_-COOH and C_60_-OH were found to be insignificant because of the large variabilities observed in the experiments, as well as the low accumulation levels.

Following i.p. exposure, C_60_-COOH was only detected in the bone, spleen and kidneys at very low concentrations, which indicated low levels of absorption in these organs. In contrast, C_60_-OH translocated into a much wider range of tissues after i.p. injection, including the stomach, kidneys, lung, intestine, blood, muscle and bone, where it showed high accumulation levels. Notably, C_60_-OH was detected in tissues both in- and outside of the abdominal cavity. In a similar manner to gavage exposure, the differences observed between C_60_-COOH and C_60_-OH following i.p. exposure were determined to be insignificant.

## Discussion

According to the experimental results described above, the surface modification of fullerene nanomaterials led to changes in their accumulation levels in specific organs. At 24 h post exposure via i.v. administration, unmodified C_60_ was found to be mainly distributed in the liver, spleen and lung, whereas C_60_-COOH and C_60_-OH were mainly trapped in liver, bone, muscle and skin following the same time period. These findings therefore implied that the chemical functionalization of C_60_ with carboxyl or hydroxyl groups resulted in the formation of materials that could be eliminated from the liver or deposited in bone with greater ease than the parent fullerene.

Low accumulation levels of C_60_-OH after i.v. administration have also been reported by several other groups. For example, Li et al. reported the biodistribution of ^67^Ga-C_60_(OH)_x_ after i.v. exposure [46]. In this particular case, the liver (12.9%ID/g), spleen (17.1%ID/g), skull (23.7%ID/g) and thighbone (17.6%ID/g) showed relatively high accumulation levels of the modified fullerene at 24 h, whereas all of the other organs showed accumulation levels of less than 4%ID/g. Using another radioactive isotope ^99m^Tc, Li et al. found that ^99m^Tc-C_60_(OH)_x_ was distributed in the liver (9.4%ID/g), spleen (7.2%ID/g), bone (8.4%ID/g), lung (1.7%ID/g), kidneys (5.2%ID/g), intestine (2.1%ID/g) and heart (0.7%ID/g) of mice with low accumulation levels at 24 h [47]. Similarly, Song et al. reported that ^99m^Tc(CO)_3_-C_60_(OH)_20_ distributed in the whole body except for the brain, with accumulation levels of less than 1.0%ID/g at 24 h [48]. Maksin et al. reported the biodistribution of radiolabeled fullerenol after i.v. exposure [49]. The results of this study revealed that fullerenol was distributed into the lung (2.1%ID), liver (20.1%ID), kidney (5.3%ID), muscle (0.16%ID/g), bone (0.59%ID/g), thyroid (0.2%ID), spleen (0.66%ID) and intestine (16%ID) at 2 h. Li et al. reported the biodistribution of C_60_(OH)_x_(O)_y_ after i.v. exposure, and showed that fullerenol was distributed into the heart, lung, liver, spleen, kidney, muscle, bone, intestine, fur and brain [50]. Furthermore, relatively high accumulation levels were found in the liver (4.3%ID/g), spleen (2.3%ID/g), kidney (4.1%ID/g) and bone (8.0%ID/g) at 24 h post exposure. Ji et al. labeled C_60_(OH)_x_ with ^125^I and followed the translocation of ^125^I-C_60_(OH)_x_ in tumor-bearing mice [51]. The results showed that ^125^I-C_60_(OH)_x_ was distributed through the whole body of the mice, except for their brain, and that relatively high accumulation levels were found in the liver (2.0%ID/g), stomach (1.2%ID/g), kidney (1.6%ID/g) and bone (1.7%ID/g) at 24 h. These results from the literature therefore confirm the reliability of our distribution results for C_60_-OH. Furthermore, based on the experimental reports described above, it is clear that the surface hydroxylation of C_60_ leads to improved water-solubility and bioavailability. These changes also enhance the ability of these materials to cross biological barriers in vivo, and therefore reduce the accumulation and retention levels of these compounds in the tissues/organs.

Furthermore, hydroxylation led to slightly lower levels of RES capture than those encountered following the carboxylation of the fullerene core. The low RES capture of C_60_-OH observed in this study was consistent with the results of several other studies. For example, ^67^Ga-, ^125^I- and ^99m^Tc-labeled fullerenols all showed low levels of RES capture [46–51]. Pristine C_60_ nanoparticles are hydrophobic xenobiotic substances, which are easily trapped in the RES organs as part of an organism’s natural defense mechanism. While surface chemical modifications can lead to enhanced hydrophilicity and biocompatibility properties in fullerene nanomaterials, as well as enhancing their ability to cross biological barriers in mice, they can also lead to reduced uptake into the RES organs.

Our biodistribution results for C_60_-COOH were different from those reported by Yamago’s group [52]. In Yamago’s study, C_60_ was functionalized via a cycloaddition reaction with dipolar trimethylenemethane to give a water-soluble fullerene derivative bearing terminal carboxyl groups. These fullerene derivatives were predominantly trapped in the liver (91.7%ID at 16 h), with the accumulation levels in the heart, spleen, lung, kidneys, brain and testicles being much lower. The difference between our results and those of Yamago’s groups could be attributed to differences in the functionalization density and the length of chains attached to the carboxylic acid groups. In our study, each fullerene cage had two carboxyl groups, which were directly linked on the pyrrolidine ring. In Yamago’s study, each fullerene cage had only one carboxyl group, which was linked to the five-membered ring with a small linker. The solubility of C_60_-COOH was therefore much higher than that of the fullerene derivatives described in Yamago’s study. The lower hydrophilicity of Yamago’s compounds would lead to higher levels of protein binding. Yamago’s fullerene derivatives would also be recognized by opsonin and more easily trapped in the RES organs.

The surface functionalization of fullerene also affected its bioavailability and distribution following the gavage and i.p. administration routes, although the differences observed in these cases were not found to be significant because of their low accumulation levels. The high absorption of C_60_-COOH following gavage exposure could be attributed to the hydrophobicity of the fullerene cage and the small size of the C_60_-COOH aggregates. There have been several reports in the literature that show that small nanoparticles with good hydrophobicity show high bioavailability after oral exposure [53–59]. However, following i.p. exposure, the tissue translocation of C_60_-OH was much more widespread and pronounced than that of C_60_-COOH. The low translocation of C_60_-COOH observed in this case could be attributed to the negative charges on the carboxylate groups and its hydrophobicity. The free translocation of hydroxylated carbon nanomaterials has also been observed for CNTs. For example, Wang’s group reported that hydroxylated CNTs were detected in the whole body after i.p. injection [60, 61]. The results of the current study suggest that the translocation and distribution properties of functionalized fullerenes are regulated by their surface-modified groups. Moreover, accumulated fullerene derivatives could be cleared from the body during long-term observations, since the i.v. injection data clearly indicated that the fullerene derivatives had been cleared completely at 7 d post exposure.

Mechanistically, how the surface regulates bioavailability and biodistribution might be related to the opsonization of fullerene. It is well known that nanoparticles entering systemic circulation can be tagged with opsonin. The subsequent recognition of opsonin can lead to these nanoparticles being captured by phagocytic cells in the RES organs. We previously demonstrated that surface functionalization can affect the interactions formed between fullerenes and proteins [62, 63]. For example, increasing the degree of functionalization (hydroxylation) led to a decrease in the extent of several fullerene–protein interactions, most likely because of the reduced opsonization of C_60_-OH. The lower accumulation levels of C_60_-COOH and C_60_-OH could therefore be attributed to reduced levels of opsonization following their surface modification.

It is noteworthy that C_60_-COOH and C_60_-OH formed different-sized aggregates because of the differences in their functionalization. The differences in the sizes of these aggregates could also affect their biodistribution behaviors. However, the influence of their size would be much less pronounced in this case, because the particulate sizes of C_60_-COOH and C_60_-OH were smaller than the pulmonary capillary diameter. When particulate size is smaller than the pulmonary capillary diameter, the particles can escape pulmonary filtration and be captured by the RES via opsonization [20, 21]. Opsonization is mainly dependent on the surface of a given nanomaterial, whereas particle size plays a much smaller role.

It is also noteworthy that the accumulation levels of C_60_-COOH and C_60_-OH found in bone and muscle (hind leg) were both relatively high following short-term observation (up to 24 h), suggesting that the total amounts of accumulation in bone and muscle were large. To estimate the total uptakes, we assumed that the accumulation levels in whole bone, muscle and skin were homogenous and that the tissue indices (tissue weight/body weight) were 10, 25 and 10% for bone, muscle and skin, respectively. The bone uptake was 25 ± 3%ID for C_60_-COOH and 36 ± 1%ID for C_60_-OH. The muscle uptake was 48 ± 21%ID for C_60_-COOH and 40 ± 3%ID for C_60_-OH. The skin uptake was 14 ± 3%ID for C_60_-COOH and 21 ± 5%ID for C_60_-OH. The percentage of C_60_-COOH increased from 42%ID at 5 min to 112%ID at 24 h, whereas the percentage of C_60_-OH increased from 45%ID at 5 min to 123%ID at 24 h. This implied that both C_60_-COOH and C_60_-OH were rapidly cleared from the blood and distributed in the interstitial fluid, from which point they gradually migrated into the organs and tissues during the first 24 h. The total percentages at 24 h were all larger than 100%ID, suggesting that while our original estimation was inaccurate, the trends (not the accurate values) were meaningful.

The results of the current study clearly show that the functionalization shifted the uptake of fullerene from the RES organs to the soft tissue and skeleton. Similar trends to this have been reported elsewhere in the literature, where the hydroxylation of fullerene led to high levels of accumulation in the bone and muscle tissues [46–51]. These differences could be attributed to the functionalized fullerenes being better equipped to escape capture by the RES and more capable of making their way into the soft tissues and bone. The retention of these fullerene derivatives in the body remained transient, with no statistically significant levels of accumulation being detected after 7 d. From a toxicological perspective, this shift in the accumulation of fullerene derivatives from the RES toward the soft tissues and bone would definitely have an impact on their toxicity. Reduced levels of RES capture would relieve the toxicity of fullerenes toward the liver, lung and spleen, although the translocation of these materials to other organs and tissues could lead to toxicity elsewhere. The good news in this case is that these fullerene derivatives were rapidly cleared from the body. The long-term toxicities of fullerene derivatives could be lower than those of the corresponding pristine materials. Future studies should therefore focus on the toxicological evaluation of pristine and functionalized fullerene systems to develop a better understanding of the toxicological impact of functionalization.

## Conclusions

In summary, we have quantitatively investigated the impact of different surface modifications and routes of administration on the biodistribution of fullerene in vivo using a ^13^C-labeled carbon cage tracer. We specifically compared the biodistribution behaviors of C_60_-COOH and C_60_-OH in mice following their exposure via three different pathways, where C_60_-COOH and C_60_-OH showed distinct distribution patterns. The surface modification of fullerene C_60_ led to a decrease in its accumulation and rate of distribution, as well as altering its target organs. These differences clearly indicated that the chemical functionalization of fullerene has a significant influence on its translocation and biodistribution. In particular, the differences between C_60_-COOH and C_60_-OH were more distinguishable following gavage and i.p. administration. Undoubtedly, specific surface modifications could lead to improvements in the biocompatibility of C_60_, by reducing its toxicity, which could be of some benefit in terms of the biomedical application of these materials. We therefore believe that our results could benefit specific biomedical applications, as well as having an impact on biosafety studies concerning other carbon nanomaterials.

## Methods

### Materials

Amorphous carbon powder of 99% ^13^C was purchased from Cambridge Isotopes Laboratories, Inc. (Andover, MA, USA). ^13^C-C_60_ was prepared using the arc discharge method according to our previously reported procedure [34, 35]. Unlabeled C_60_ was obtained from Nanjing XFNANO Materials Tech Co., Ltd (Nanjing, China). All of the other chemicals used in this study were purchased as the analytical degree from various chemical suppliers.

### Preparation of ^13^C-C_60_-COOH and ^13^C-C_60_-OH

The carboxylation of ^13^C-C_60_ was conducted according to a modified version of the method reported by Gan et al [64]. Briefly, Me_2_HNCH_2_COOEt•HCl (279 mg, 2.0 mmol) and sodium hydroxide (228 mg, 5.7 mmol) were added to 15 mL of methanol, and the resulting mixture was stirred until it formed a solution. This solution was then added to a solution of ^13^C-C_60_ in toluene (60 mL), and the resulting mixture was irradiated with a nondestructive xenon lamp (300 W) under continuous stirring for 1 h, during which time the solution changed color from purple to brown. The solution was then concentrated under reduced pressure to approximately 20 mL and treated with sodium hydride (1 g), before being heated at 70 °C under continuous stirring for 6 h. The mixture was then cooled to room temperature and added to 10 mL of methanol before being evaporated to dryness under reduced pressure to give a residue. The residue was then added to water (50 mL) to dissolve the ^13^C-C_60_-COOH, and the resulting suspension was centrifuged (3000 × *g*, 5 min). The supernatant was then collected and purified by column chromatography over Sephadex G-25 gel to separate the desired ^13^C-C_60_-COOH material from any unwanted small molecules. The first fraction was collected and lyophilized to give ^13^C-C_60_-COOH.

The hydroxylation of ^13^C-C_60_ was conducted according to a procedure from the literature [65–67]. Briefly, a toluene solution of ^13^C-C_60_ was vigorously stirred with powdered NaOH and 50% tetrabutylammonium hydroxide (TBAH) at room temperature in an open vessel for 24 h. The biphasic reaction mixture was then separated and the aqueous phase was filtered to remove trace quantities of water-insoluble residues. The filtrate was then added with methanol, which resulted in the formation of a brown precipitate. The methanol was subsequently removed in vacuo to give a residue, which was dissolved in water and purified by column chromatography over Sephadex G-25 gel (1 × 30 cm) eluting with neutralized water. Fractions containing the desired product were freeze-dried to give ^13^C-C_60_-OH as a yellow powder.

The ^13^C-C_60_-COOH and ^13^C-C_60_-OH samples generated above were characterized by IRMS (ThermoFisher Delta V advantage; Waltham, MA, USA), TEM (Tecnai G2 F20 U-TWIN; Hillsboro, Oregon, USA), Nanosizer analysis (Nanobrook-Omni, Bruker; Holtsville, New york, USA), IR spectrometry (Mir-IR, Nicolet iN10 MX, ThermoFisher; Waltham, MA, USA) and XPS (Thermo Scientific ESCALab250Xi; Waltham, MA, USA).

### Animal administration

Animal experiments were performed humanely in accordance with the Animal Care and Use Program Guidelines of the Sichuan Province, China. Male ICR mice (25 g) were obtained from Sichuan University Animal Center, Chengdu, China. The mice were raised in plastic cages (three mice/cage) on a 12-h light/dark cycle with *ad libitum* access to food and water. Following acclimation, the mice were randomly divided into 15 groups (three mice for each group) for the biodistribution studies.

### Biodistributions of ^13^C-C_60_-COOH and ^13^C-C_60_-OH after i.v. injection

The mice were injected via their tail vein with ^13^C-C_60_-COOH or ^13^C-C_60_-OH at a single dose of 400 μg in 200 μL of 0.9% aqueous NaCl solution. Blood samples were collected periodically from the mice, which were subsequently scarified at designated time intervals. Several tissue samples, including heart, liver, spleen, stomach, kidney, lung, brain, large intestine, small intestine, bone (hind leg), muscle (hind leg) and skin (abdomen), were collected from the mice and dissected, homogenized and lyophilized before being analyzed by IRMS using our previously reported procedure [35]. The control animals were injected with 200 μL of 0.9% aqueous NaCl solution and sampled in the same way. The results of these analyses have been presented as %ID/g values for all of the tissue samples, as well as %ID values for the heart, liver, spleen, stomach, kidneys, lungs, brain, large intestine and small intestine samples.

### Translocation profiles of ^13^C-C_60_-COOH and ^13^C-C_60_-OH after gavage and i.p. administration

For gavage exposure, the mice were exposed to ^13^C-C_60_-COOH or ^13^C-C_60_-OH at a single dose of 400 μg in 200 μL of 0.9% aqueous NaCl solution via gavage. For i.p. exposure, the mice were intraperitoneally injected with ^13^C-C_60_-COOH or ^13^C-C_60_-OH at a single dose of 400 μg in 200 μL of 0.9% aqueous NaCl solution. The control groups were exposed to 200 μL of 0.9% aqueous NaCl solution according to the same protocols as those described above. All of these animals were sampled in the same way as those subjected to the i.v. injection protocol.

### IRMS analyses

The IRMS measurements and calculations were performed as follows. The IRMS system was calibrated using a standard urea sample (traceable to primary isotopic certified reference materials issued by the International Atomic Energy Agency, Vienna), because Vienna Pee Dee Belemnite (VPDB) was too expensive and only available in limited amounts. The calibration was satisfied when the value determined using our system matched that of the certified value. Whole organs or tissue samples obtained from the ^13^C-fullerene exposed mice were dissected and weighted. The liver and intestinal samples, which were too large to fit into the homogenizer, were cut into small pieces (smaller than 1 mm in diameter) with scissors and 0.5-g samples of these materials were mixed with 2.0 mL of water. All of the other organ/tissue samples were less than 0.5 g in weight and could therefore be added directly to 2.0 mL of deionized water. The samples were then thoroughly homogenized in a homogenizer. The homogenates were subsequently lyophilized to obtain dry powders, which were analyzed by IRMS. The IRMS results were obtained in the form of *δ* values, which were subsequently converted into ^13^C/^12^C ratios (*r*) following Equation 1. [(^13^ *C*/^12^ *C*)_standard_] was the ^13^C/^12^C ratio of the VPDB sample (0.0112372). Because VPDB is classical for IRMS, the calculation still refers to VPDB in the literature, where the calculating results would not be interfered by the different calibration references [36, 37]. The *r* values were then converted into percentages of ^13^C in mass (${\omega}_{13_C}$) using Equation 2, which provided the ratio of “total weight of ^13^C atoms/total weight of carbon atoms”. The mass of ^13^C-enriched fullerene derivatives in each tissue (${m}_{13_{C- fullerene}}$) was determined using Equation 3, where the *ω*_*carbon of tissue*_ value determined by IRMS was the mass percentage of carbon in the dry tissue samples from the exposed and unexposed mice (the value was constant for certain organs); *m*_*tissue*_ was the weight of tissues; *m*_*dry*_/*m*_*wet*_ was the weight ratio of the tissue samples before and after drying, which was measured using unexposed mice and was constant for certain organs; ${\omega}_{13_C}(tissue)$ was the mass percentage of ^13^C in each tissue sample from the exposed mice; ${\omega}_{13_C}(control)$ was the percentage of ^13^C in mass for tissue samples from the unexposed mice; and ${\omega}_{13_{C- fullerene}}$ was the percentage of ^13^C in the ^13^C-enriched fullerene derivatives. The content of each ^13^C-enriched fullerene derivative was expressed as %ID/g (Equation 4) or %ID (Equation 5), where the dose was equal to the mass of injected ^13^C-enriched fullerene derivative.

1 $$ r=\left(\frac{\delta }{1000}+1\right)\times {\left({}^{13}C{/}^{12}C\right)}_{\mathrm{standard}} $$

2 $$ {\omega}_{13_C}=\frac{r\times 13}{r\times 13+12}\times 100\% $$

3 $$ {m}_{13_{C- fullerene}}=\frac{\left[{\omega}_{13_C}(tissue)-{\omega}_{13_C}(control)\right]\times \left({\omega}_{carbon\kern0.5em  of\kern0.5em  tissue}\times {m}_{tissue}\times \frac{m_{dry}}{m_{wet}}\right)}{\omega_{13_{C- fullerene}}} $$

4 $$ \% ID/g=\frac{m_{13_{C- fullerene}}}{dose}\times 100\%\div {m}_{tissue\kern0.5em  sample} $$

5 $$ \% ID=\frac{m_{13_{C- fullerene}}}{dose}\times 100\% $$

### Statistical analysis

All data have been expressed as the mean values of three individual observations together with the associated standard deviation (mean ± SD). Significant differences between the biodistribution data of C_60_-OH and C_60_-COOH were calculated using the Student’s *t*-test. Differences were considered significant for *p* < 0.05. Comparisons were made in a time-dependent manner between the different exposure pathways. The %ID data of ^13^C-C_60_-OH and ^13^C-C_60_-COOH were compared with those of the unmodified C_60_ to reveal the impact of the surface functionalization on the biodistribution of fullerene.

## Abbreviations

%ID: percentage of injected dose
%ID/g: percentage of injected dose per gram tissue
CNTs: carbon nanotubes
i.p.: intraperitoneal
i.v.: intravenous
IR: infrared
IRMS: isotope ratio mass spectrometry
RES: reticuloendothelial system
TEM: transmission electron microscopy
VPDB: Vienna Pee Dee Belemnite
XPS: x-ray photoelectron spectroscopy
