# Erratum to: Surface modification-mediated biodistribution of ^13^C-fullerene C_60_ in vivo

**DOI:** 10.1186/s12989-016-0153-5

**Published:** 2016-08-17

**Authors:** Chenglong Wang, Yitong Bai, Hongliang Li, Rong Liao, Jiaxin Li, Han Zhang, Xian Zhang, Sujuan Zhang, Sheng-Tao Yang, Xue-Ling Chang

**Affiliations:** 1 Northwest University, Xi’an, 710069 P. R. China; 2 CAS Key Laboratory for Biomedical Effects of Nanomaterials and Nanosafety, Institute of High Energy Physics, Chinese Academy of Sciences, Beijing, 100049 P. R. China; 3 College of Chemistry and Environment Protection Engineering, Southwest University for Nationalities, Chengdu, 610041 P. R. China; 4 Key Lab of Urban Environment and Health, Institute of Urban Environment, Chinese Academy of Sciences, Xiamen, 361021 P.R. China

## Erratum

After the publication of this work [1] it was noticed that the captions for Figures 4 and 5 were accidentally swapped.

The original article has been updated.
